# Optimized Inner-Volume 3D TSE for High-Resolution Vessel Wall Imaging of Intracranial Perforating Arteries at 7T

**DOI:** 10.3389/fnins.2021.620172

**Published:** 2021-02-25

**Authors:** Qingle Kong, Yue Wu, Dehe Weng, Jing An, Yan Zhuo, Zihao Zhang

**Affiliations:** ^1^State Key Laboratory of Brain and Cognitive Science, Beijing MR Center for Brain Research, Institute of Biophysics, Chinese Academy of Sciences, Beijing, China; ^2^MR Collaboration, Siemens Healthcare Ltd, Beijing, China; ^3^University of Chinese Academy of Sciences, Beijing, China; ^4^Innovation Center for Excellence in Brain Science, Chinese Academy of Sciences, Beijing, China; ^5^Siemens Shenzhen Magnetic Resonance Ltd, Shenzhen, China

**Keywords:** vessel wall imaging, perforating arteries, 7T, high-resolution, inner-volume imaging

## Abstract

The impairment of microvessels can lead to neurologic diseases such as stroke and vascular dementia. The imaging of lumen and vessel wall of perforating arteries requires an extremely high resolution due to their small caliber size. Current imaging techniques have the difficulty in observing the wall of perforating arteries. In this study, we developed a 3D inner-volume (IV) TSE (SPACE) sequence with optimized 2D spatially selective excitation (SSE) RF pulses. The optimized SSE RF pulses were designed through a series of optimization including iterative RF pulse design, trajectory optimization, and phase convention of Carr-Purcell-Meiboom-Gill (CPMG) condition to meet the perforating arteries imaging demands. High resolution of isotropic 0.30 mm within 10 min was achieved for the black- blood images of lenticulostriate artery (LSA). The LSA lumen and vessel wall were imaged by the IV-SPACE sequence simultaneously. Images obtained by the optimized RF pulse has fewer aliasing artifacts from outside of ROI than the traditional pulse. The IV-SPACE images showed clearer delineation of vessel wall and lumen of LSA than conventional SPACE images. IV-SPACE might be a promising method for detecting microvasculopathies of cerebral vascular diseases.

## Introduction

Lacunar infarction (LI) may be caused by lipohyalinotic small artery disease (SAD) (Fisher, 1982), atherosclerotic SAD (Caplan, 1989), and the occlusion of perforators because of parental artery atherothrombosis (Bang et al., 2002). Distinguishing the causes of LI is essential for potential guiding therapeutic intervention. Unfortunately, current imaging techniques cannot differentiate atherosclerotic from lipohyalinotic SAD (Kim and Yoon, 2013) due to the difficulty in observing the wall of small arteries. Visualization of the vessel wall of small arteries (such as perforating artery) requires extremely high spatial resolution and signal-to-noise ratio (SNR) due to their small caliber size and slow flow.

According to previous radiological studies (Mandell et al., 2017), the following features were required for vessel wall imaging (VWI) of intracranial arteries: high spatial resolution, sufficient SNR, suppression of signal in luminal blood and CSF, and multiple tissue weightings. To meet these requirements, turbo spin echo (TSE) and its variants become the most widely used MR sequence for intracranial VWI (Van der Kolk et al., 2011; Zhu et al., 2016; Harteveld et al., 2017). And for 3D acquisition, TSE sequence with variable flip angle (named SPACE by Siemens) is usually used to obtain isotropic high-resolution images to reduce partial volume effects (Fan et al., 2017). Qiao et al. used 3D TSE to obtain 0.4 mm isotropic images to evaluate intracranial vessels at 3.0T (Qiao et al., 2011), which is the highest resolution currently reported for 3D VWI. The SPACE at 7T has higher SNR and intrinsic black blood characteristics, which is very suitable for high-resolution VWI.

Nevertheless, the isotropic 0.40 mm voxel is not sufficient to delineate the vessel wall of intracranial small arteries, of which the thickness was 0.05–0.40 mm. To visualize the thinner vasculature, the spatial resolution of VWI must be further improved. However, conventional 3D TSE employed hard pulse for excitation and required whole-brain acquisition to avoid image aliasing. As a result, high spatial resolution increased phase-encoding (PE) steps in 3D TSE, which means four times the acquisition time (TA) when the dimension of a voxel is halved.

Theoretically, TA can be shortened by increasing acceleration factors. However, too aggressive down-sampling further impaired the limited SNR of small voxels and downgraded the quality of displaying tiny structures in images. The inner volume imaging (IVI) was another way to reduce the PE steps and shorten TA. This can be achieved by limiting the excitation area to reduce the field of view (FOV) (Feinberg et al., 1985). To obtain inner volume selection, orthogonal excitation in combination with refocusing pulses (Feinberg et al., 1985), or 2D spatially selective excitation (SSE) radio frequency (RF) pulses can be used (Pauly et al., 1989). SSE was particularly suited for IVI using 3D TSE sequences because magnetization not tipped by the initial excitation pulse did not generate signal at any of the subsequent echo times so long as sufficient spoiling was included to suppress free induction decay (FID) signals produced by refocusing pulses.

Previous work has verified the feasibility of combining 2D RF pulse and 3D TSE sequence (Mitsouras et al., 2006). However, because it is a preliminary study, some limitations still exist and hinder its practical application, such as incomplete background suppression and long RF pulse durations. Background suppression is critical for inner-volume imaging, particularly when selecting a small sub-volume since the volume contributing undesired background signals is large. In addition, the RF designed method that previous work used is non-ideal in minimizing excitation error. This work designed the SSE RF pulses successfully to meet the perforating arteries (especially lenticulostriate artery, LSA) imaging demands through a series of optimization including iterative RF pulse design, trajectory optimization, and phase convention of Carr-Purcell-Meiboom-Gill (CPMG) condition (Meiboom and Gill, 1958). The optimized 2D SSE RF pulses were used to replace the excitation pulses of the conventional SPACE sequence. Inner volume (IV) imaging of SPACE (IV-SPACE) was achieved to reduce the acquisition matrix and obtain high-resolution VWI images of LSA. The LSA lumen and vessel wall were imaged by the IV-SPACE sequence with black-blood 0.30 mm isotropic resolution. The imaging results from the optimized RF pulse were compared to the traditional 2D RF pulse. The LSA vessel wall imaging from the IV-SPACE sequence was also compared with conventional SPACE.

## Materials and Methods

### Subjects

With the approval of the local institutional review board, eleven healthy volunteers were recruited and scanned. Four of the subjects were male, seven females, and the average age was 26 (± 2) years. Written informed consent was obtained from all subjects.

### MRI System

All scans were performed on a whole-body human 7T MR research system (Siemens Healthcare, Erlangen, Germany), with a maximum gradient amplitude of 70 mT/m and maximum gradient slew rate of 200 mT/m/ms. A 32-channel head coil was used for signal reception at 7T.

### 2D Spatially Selective RF Pulse Design

**FIGURE 1:**
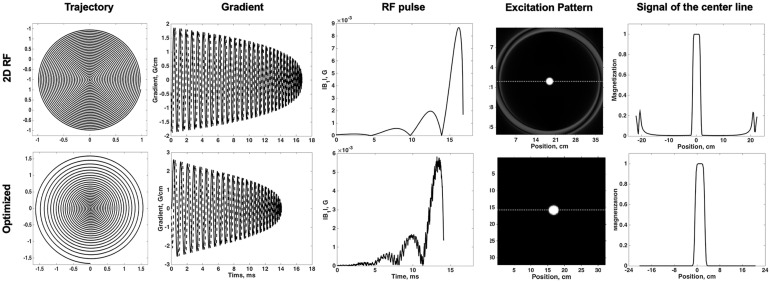
Comparisons of the traditional 2D SSE pulse (top row) and the optimized method (bottom row): trajectory, gradient, RF pulse, excitation pattern in the excitation plane, and the signal of the center line.

The traditional 2D SSE RF pulse (FA = 90°) was designed with a constant angular rate spiral-in transmit k-space trajectory, 32 turns, and a pulse length of 16.75 ms (Figure 1). The calculation method was described in Pauly et al. (1989). The disk diameter was 5 cm, and the excitation FOV was 17 cm to avoid excitation aliasing. These parameters were selected based on the anatomical distribution of the middle cerebra artery (MCA) and LSA. The Bloch simulation was performed to verify the performance of SSE RF pulse and phase relaxation and off-resonance were ignored.

To design the optimized RF pulse that excited the desired 2D profile, B_0_ and B_1_ information together with the subject’s geometry were needed. Therefore, maps of the local B_0_ offsets (echo time (TE) difference: 2.3 ms, scan time: 18 s) and B_1_ efficiency [dual repetition time (TR) method (Insko and Bolinger, 1993), TR: 80 ms, scan time: 74 s] were acquired of a sagittal slice in the brain. A mask indicating the subject’s brain was obtained from the threshold of the first echo image of the B_0_ map. The position of the LSA in the sagittal plane was determined from sagittal localizer images.

Variable-density spiral trajectories were used to under-sample k-space regionally, thus reducing pulse length. The variable-density spiral-in trajectory within hardware limitations of the gradient system was designed to adequately sampled excitation k-space near the origin (XFOV = 25 cm), and under sampled in the high-frequency region (XFOV = 6 cm) (Figure 1). The optimized SSE RF pulses (FA = 90°) were calculated following the method described in Yip et al. (2005) and using the natural suppression mechanism of the CPMG condition. The feature of TSE sequences is that excitation and refocusing pulses must rotate the magnetization vector around orthogonal axes. If this condition is not met, echo amplitudes rapidly decrease when the refocusing angle is not precisely 180° (Meiboom and Gill, 1958). Therefore, the component of the excited magnetization that is perpendicular to the rotation axis of the refocusing pulses (i.e., the non-CPMG component) will yield echoes that quickly die away through the imaging sequence. If long echo trains are used with linear phase encode ordering the resulting signals are strongly suppressed. This work achieved this enhanced background suppression by performing a weighted least squares minimization like the methods proposed by Malik and Hajnal (2016). Weighting factor wr = 0.25 and regularization parameter λ = 100 was used for RF pulse designs based on pilot data performance. A typical example was shown in Figure 1. The pulse length was 14.10 ms. The delay time between gradient and RF pulse was corrected in the scanner. Individual pulse design processes took approximately 1 min using MATLAB (R2017b, The MathWorks, Inc., Natick, MA, United States) on a computer with a 3.1 GHz dual core processor and 8 GB RAM.

### Experiment

A phantom experiment was performed to explore the performance of the traditional 2D RF pulse and the optimized RF pulse. The phantom was a sphere filled with a solution of NiSO_4_ (T_1_/T_2_: 420 ms/240 ms). The IV-SPACE for phantom imaging had field of view (FOV) 190 mm × 190 mm, TR = 1500 ms, TE = 10 ms, Matrix = 144 × 144 × 128, Resolution = 1.3 ×1.3 ×1.3 mm^3^, ETL = 50, TA = 2 min 5 s.

The effect of the 2D SSE pulses (two methods) was also assessed on a low resolution (1.8 ×1.8 ×1.8 mm^3^) scan of the entire brain (scan time = 3 min 11 s). The local higher resolution scan of the MCA region was performed subsequently. The scan parameters were as follows: TR = 1500 ms, TE = 10 ms, Matrix = 848 ×212 ×192, Resolution = 0.3 ×0.3 ×0.3 mm^3^, ETL = 30, Average = 1.6, GRAPPA = 3, TA = 9 min 57 s. High-resolution imaging was performed for both two pulses. In order to evaluate the LSA vessel wall of the IV-SPACE sequence, a 0.4 mm isotropic conventional SPACE data acquisition was also performed in this study. The detailed imaging parameters can be found in previous work (Kong et al., 2019).

### Data Analysis

To quantitatively assess the degree of suppression outside of ROI which optimized method achieved, the suppression ratio was calculated for both pulses. The suppression ratio was calculated as the ratio of the average signal inside a 1 cm^2^ volume within the excited inner volume to the outer volume region. In order to compare the uniformity of the signal in ROI, the ratio of standard deviation and magnitudes within the ROI was calculated for both two methods. The line profile through the excitation center was also extracted and normalized to quantitatively assess the performance of the optimized method.

Coronal MinIP (projection thickness = 18 mm) was generated with VWI images of all three methods. When generating the MinIP, the center of the slab was adjusted until the maximal number and the longest visible length of LSAs were obtained. The number of stems and branches were counted by two independent readers (Z.H.Z., Q.L.K.; 5 and 3 years of experience in VWI, respectively). Stems were defined as the LSAs that originated directly from the MCA M1, and branches were defined as daughter vessels originating from the parent LSA stems plus stems without any branches. The signal-to-noise ratio (SNR) was also calculated for all three methods. The ROI of signal was obtained from the brain tissue adjacent to LSA (circle, diameter = 5 mm). The noise was estimated as the standard deviation of the ROI (circle, diameter = 5 mm) at the margin of images without brain tissue. The ROIs were kept consistent in the images acquired by all three methods.

To demonstrate the feasibility of using IV-SPACE to visualize the LSAs vessel wall, curved multi-planar reconstruction (curved-MPR) was constructed along the MCAs and transverse minimum intensity projections (MinIP, projection thickness = 2 mm) was generated with VWI images from both IV-SPACE and conventional SPACE. The section view of LSA was built perpendicular to the center line of LSA so that the vessel wall of LSAs could be readily identified in the plane. A cut line through the vessel center was extracted and normalized to compare the wall characterization of two pulses. All the analysis was executed in OsiriX (Rosset et al., 2004).

All quantitative data were expressed as means ± standard deviations. Wilcoxon signed-rank test was used to compare the numbers of stems and branches between the pairwise comparison of the three imaging methods. A *p*-value of less than 0.05 indicated statistical significance. The intraclass correlation coefficient (ICC) was calculated and reported for the measurements of LSA. All statistical analyses were performed using commercial software (SPSS 22.0, IBM).

## Results

**FIGURE 2:**
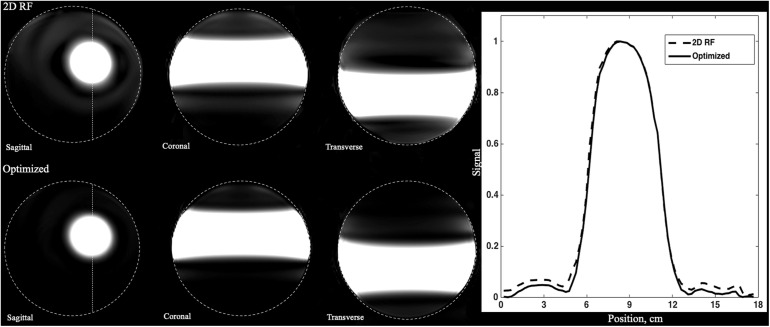
Comparison of the phantoms excited by traditional SSE RF pulse and optimized pulse. Images were shown in the same window-level which was increased the to show the signal residuals outside the ROI. The optimized RF pulse exhibits less residual signal in the outer-volume, which is most obvious in sagittal plane. The line profile through the center of ROI was shown in right column. The solid line is for the optimized pulse and the dashed line is the traditional pulse. Optimized RF pulse has better signal suppression outside the ROI and fewer side lobes.

The Bloch simulation of the traditional 2D RF pulse and optimized RF pulse were shown in Figure 1. The simulation results showed that the designed SSE pulse achieved inner volume selection successfully. The phantom results of the excitation profile for two methods were shown in Figure 2. They were consistent with the simulation results. The images were set to have the same window level and to show the residual signal outside the ROI as much as possible. Images in Figure 2 showed that the optimized RF pulse had less residual signal outside the ROI. The line profile through the excitation center was also extracted and normalized, as shown in the right column of Figure 2. The average signal outside the ROI of the image obtained by optimized RF pulse was 2%, and by traditional 2D RF pulse was 8%, which further showed that the optimized RF pulse had better signal suppression outside the ROI and fewer side lobes. The line profile before the normalization was shown in Supplementary Figure 1.

In order to compare the uniformity of the signal in ROI, the ratio of standard deviation and magnitudes within the ROI was calculated for both pulses. The ratio within ROI of the optimized RF pulse was 0.0016, while the 2D RF pulse was 0.0021. Obviously, the optimized RF pulse had a better uniformity in ROI, which may be due to the better correction of B_1_ and B_0_. The widths of the transition band of the phantom images obtained by the two pulses were similar, accounting for about 25% (1.25 / 5) of the pass bandwidth.

**FIGURE 3:**
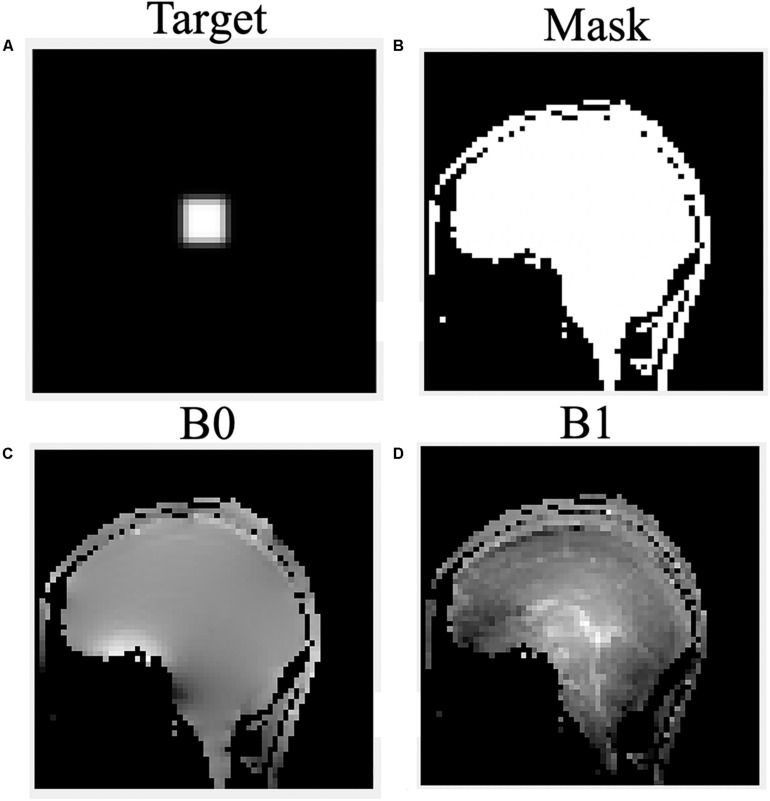
The desired pattern **(A)**, mask **(B)**, B_0_ **(C)**, and B_1_ **(D)** field map for optimized RF pulse design on a volunteer.

**FIGURE 4:**
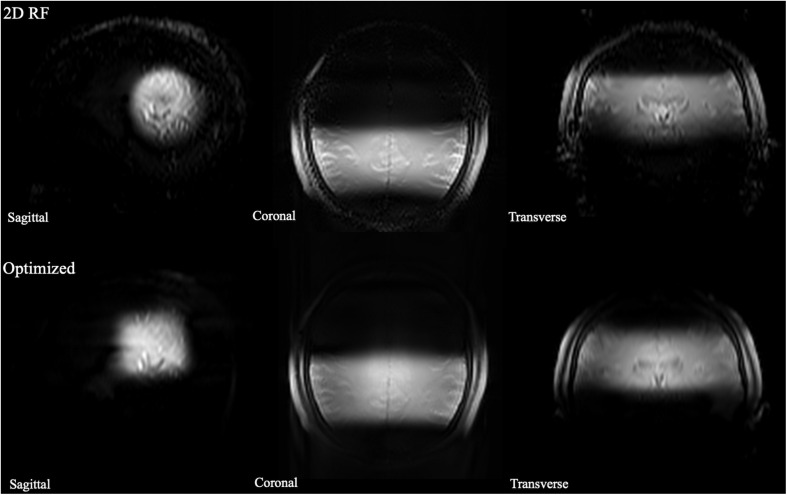
The comparison of full-FOV IV-SPACE images using different RF pulse designs. The upper is from a traditional SSE RF pulse and the bottom is from an optimized pulse. Optimized RF pulse has better suppression of signals outside the ROI.

The B_0_ and B_1_ maps obtained from a volunteer for optimized RF pulse design were shown in Figures 3C,D. The desired target was 5 cm ×5 cm (Figure 3A). The mask obtained by threshold processing accurately depicted the brain contour of subjects (Figure 3B), which further improved the excitation accuracy. The typical excitation pattern of the 2D SSE pulses designed by the optimized method and the traditional method was shown in Figure 4, in a low-resolution scan across the entire brain. A large signal intensity difference between the ROI and the rest of the ROI can be observed. Figure 4 showed that the optimized RF pulse had better suppression of signals outside the ROI, which was especially important for local high-resolution imaging. The more thorough signal suppression outside the ROI, the smaller the aliasing artifacts in the ROI of the high-resolution image.

**FIGURE 5:**
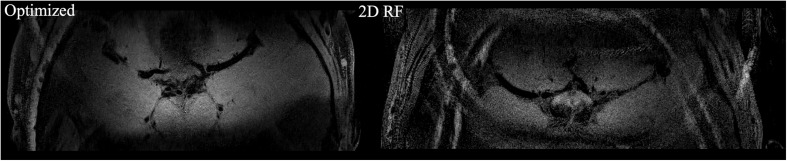
The comparison of high resolution IV-SPACE images using a traditional 2D SSE RF pulse and an optimized RF pulse. Images obtained by the optimized RF pulse has fewer aliasing artifacts from the outer volume than the traditional pulse.

The high-resolution 0.3 mm isotropic images obtained by the optimized RF pulse and the traditional 2D RF pulse were shown in Figure 5. Similar to the low-resolution results, the images obtained by the optimized RF pulse had fewer aliasing artifacts from outside of ROI, making it easier to observe the LSA vessel wall.

Finally, ten datasets were used for analysis (one was removed due to head motion). The numbers of stems and branches of the LSAs are summarized in Table 1. The *p* values of Wilcoxon signed-rank test on SNR and the numbers of stems and branches listed in Table 2. The numbers of stems visualized among three different methods were comparable. The number of branches visualized by 2D RF and conventional SPACE were comparable, and both them were lower than that by optimized method (Optimized vs. Conventional SPACE = 7.8 ± 1.54 vs. 7.1 ± 1.33, *p* = 0.002, ICC = 0.817; 2D RF vs. Optimized = 7.0 ± 1.78 vs. 7.8 ± 1.54, *p* = 0.001, ICC = 0.805). Optimized methods yielded a better SNR than 2D RF (*p* = 0.017), and both these two methods were significantly lower than conventional SPACE (Optimized vs. Conventional SPACE: *p* = 0.001, 2D RF vs. Conventional SPACE: *p* < 0.001).

**TABLE 1:** The numbers of stems and branches of LSAs and SNR among the three methods.

	Stem	Branch	SNR
Conventional SPACE	5.1 ± 0.85	7.1 ± 1.33	80.7 ± 23.36
2D RF	5.0 ± 0.86	7.0 ± 1.78	46.7 ± 5.08
Optimized	5.2 ± 0.87	7.8 ± 1.54	53.3 ± 8.57

**TABLE 2:** The comparison of the numbers of stems, branches, SNR among conventional SPACE, 2D RF and optimized methods (*p < 0.050, **p < 0.005).

	Stem		Branch		SNR
	*p* value	ICC	*p* value	ICC	*p* value
Optimized vs. 2D RF	0.186	0.858	0.001**	0.805	0.017*
Optimized vs. conventional SPACE	0.330	0.926	0.002**	0.817	0.001**
2D RF vs. conventional SPACE	0.163	0.847	0.681	0.849	0.000**

**FIGURE 6:**
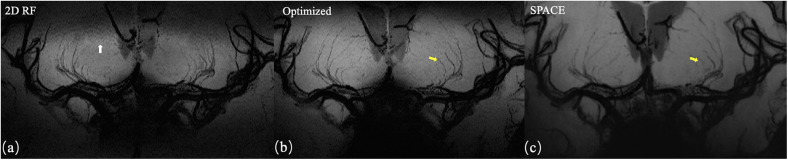
Coronal MinIP (projection thickness = 18 mm) images of 2D SSE pulse **(a)**, optimized method **(b)**, and conventional SPACE **(c)**. The optimized method achieves better signal suppression compare to traditional 2D RF and exhibits fewer aliasing artifacts (white arrow). Due to the higher spatial resolution, the optimized method shows more branches that are invisible in conventional SPACE images (yellow arrows).

The representative coronal MinIP images of a volunteer were shown in Figure 6 for 2D RF (a), optimized method (b), and conventional SPACE (c). The optimization method achieves more thorough signal suppression compare to traditional 2D RF and suffers from fewer aliasing artifacts (white arrow). Benefiting from the advantages of increased spatial resolution, the optimized method shows more branches that are invisible to conventional SPACE images (yellow arrows).

**FIGURE 7:**
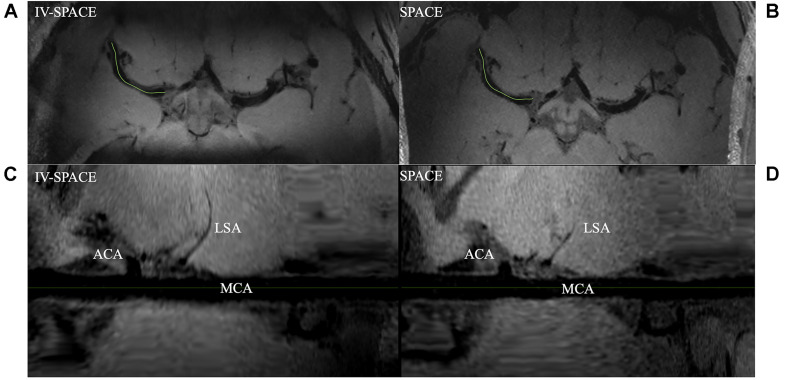
Comparison of IV-SPACE and conventional SPACE in displaying LSA. The enlarged images at the bottom are curved-MPR results along the right MCA [the green lines in **(A,B)**] of IV-SPACE **(C)** and conventional SPACE **(D)**. The lumen of an LSA in the IV-SPACE image is clearly depicted. The visible length of the LSA is longer than that in conventional SPACE.

Axial images and curved multi-planar reconstruction along the MCA were shown in Figure 7. The lumen of an LSA in the IV-SPACE image is clearly depicted and longer than conventional SPACE.

**FIGURE 8:**
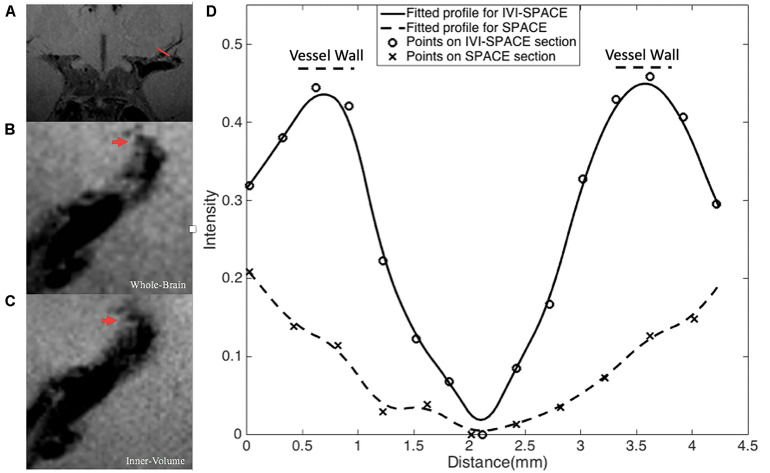
The section view of an LSA that is only visible in the IV-SPACE image. The position of the section was at the red line in coronal view **(A)**. The sections of conventional SPACE and IV-SPACE are shown in **(B,C)**, respectively. The line profiles through the LSA lumen are also shown in **(D)**.

**FIGURE 9:**
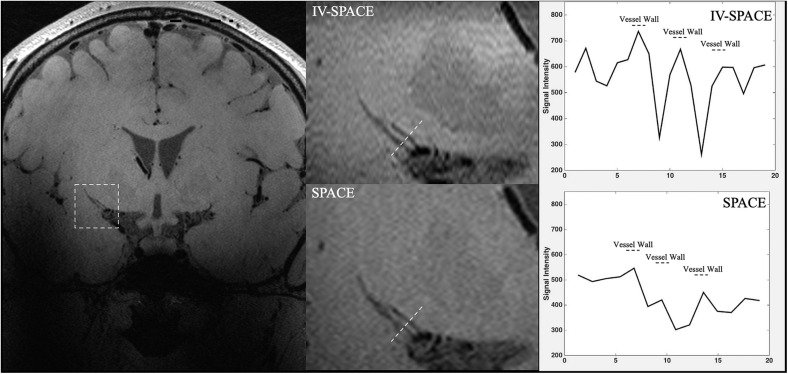
The comparisons of IV-SPACE and conventional SPACE (using optimized RF pulse) in coronal plane. The enlarged image is displayed in the second column and line profiles through the LSA lumen is shown on the right column.

In Figure 8, the section view of an LSA was shown and analyzed. The LSA vessel wall was obvious in IV-SPACE (red arrow) but almost invisible in the conventional SPACE image. The increased spatial resolution of the IV-SPACE image can be appreciated in the sharper depiction of the LSA vessel wall as compared with the conventional SPACE image. With a cut line through the vessel center, IV-SPACE images showed a significant signal drop at the lumen, which was almost absent in conventional SPACE images. Figure 9 showed another example that the lumen and orifice of an LSA were clearly depicted in the IV-SPACE image, whereas the lumen and orifice were blurred in conventional SPACE. On the line profile extracted by IV-SPACE, the lumen and wall of the two LSAs could be clearly identified, but the signal contrast of the wall to the lumen in SPACE was weak. The wall-to-lumen signal ratio of the LSA for IV-SPACE was 2.6, and the ratio for conventional SPACE was 1.5. The contrast between the vessel wall and lumen for LSA in IV-SPACE was better than conventional SPACE.

## Discussion

Although optimized forms of 3D TSE imaging have become available, the adoption of 3D sequences into routine clinical imaging has been limited primarily by long image acquisition times. Long acquisition time also hinders its high-resolution applications. Inner volume methods offer a potential solution to this limitation by combining multidimensional RF excitations to confine coherent transverse magnetization within a given volume of interest (Alley et al., 1997; Yang et al., 1999). This enables reduced FOV imaging of an anatomic region without aliasing artifacts and with significantly reduced acquisition times or achieve high resolution. In this study, the SSE RF pulse is combined with the SPACE sequence to selectively excite the basal ganglia region where MCA and LSA are located, thereby reducing the imaging FOV and reducing the size of the sampling matrix. Multiple scans are used to improve the image SNR after increasing the scanning speed. This achieves local high-resolution LSA vessel wall imaging.

This work successfully produced a clear vessel wall image of the proximal LSA. This is the first time intracranial black-blood images with isotropic 0.30 mm resolution within 10 min have been demonstrated. The optimized SSE RF pulse is robust, and no obvious signal folding from outer excitation FOV is observed. The results demonstrate that IV-SPACE imaging of the LSA vessel wall is feasible in a population of healthy volunteers.

The numbers of stems of LSAs visualized by three methods showed no significant difference, demonstrating comparable abilities of these methods on displaying primary structures of LSAs. The higher number of branches of optimized methods may be due to the higher resolution reveals much more details. The lower number of branches in 2D RF methods may be due to interference of aliased signals caused by incomplete signal suppression outside ROI (Figure 6, white arrows). The optimization method achieves more thorough signal suppression and shows more details and more branches. Of course, it must be realized that the acquisition of higher resolution comes at the expense of the signal-to-noise ratio. However, the higher resolution at the expense of the SNR seems to be beneficial, because more branches are revealed, and the sacrifice of the SNR does not cause much damage to the image quality.

Conventional SPACE uses a non-selective pulse with whole-brain coverage. Slab-selective SPACE reduces the matrix size only in the head-foot direction, while the longer duration of the excitation pulse prolongs the occurrence of the first echo and reduces the SNR. In contrast, the optimized 2D SSE RF pulse reduces FOV in both the anterior-posterior and head-foot directions. Therefore, IV-SPACE needs a smaller acquisition matrix to cover MCA and LSA than conventional SPACE to achieve the same resolution. IV-SPACE can be used to reduce acquisition times or have a higher resolution. Moreover, the inherently long and symmetric slab-selective pulse requires an extra 180° pulse as the first echo to reduce the subsequent echo spacing. However, it is not needed with a spiral-in 2D SSE pulse, resulting in a stronger signal that is suitable for a perforating artery image.

A higher spatial resolution reduces the partial volume effect, so the LSA delineation is significantly improved in the IV-SPACE images in Figure 7. The reduction of the echo train length from 50 to 30 might also contribute to the sharpness of the vessel walls due to less signal decay (Figure 8). The sharper vessel wall potentially benefits the diagnosis and also the study of cerebral vascular diseases, such as the microvasculopathy in small vessel disease, quantitative evaluation of the aneurysmal wall, and microstructure of atherosclerotic plaques.

The initial results reported herein are encouraging as they demonstrate IV-SPACE to be a robust, reliable technique for imaging of LSA wall. Using the inner volume technique, it is possible to excite a small FOV without aliasing artifacts, thus yielding high-quality imaging within the selected volume of interest. As zoomed imaging reduces the acquisition matrix while maintaining acquisition time, higher spatial resolution (not previously implemented) is achieved. Thus, IV-SPACE imaging may become the preferred approach for VWI of LSA.

The work that can be done in the future is to extend the IV-SPACE sequence to 3T scanners. Due to the popularity of 3T scanners in the clinical environment, the IV-SPACE sequence may be widely used and may exhibit more advantages. The better homogeneity of the magnetic field at 3T (Ma et al., 2019) benefits the observation of the perforating arteries originating from the M2 segment, and the lower SAR value at 3T relaxed the restrictions on sequence parameters of the IV-SPACE. But the challenge of 3T is that the SNR is only half of that at 7T, which may hinder the depiction of the LSA wall clearly. Although the nominal resolution can be set to 0.30 mm, it certainly needs further improvement on the imaging sequence to compensate for the low SNR.

This study has several limitations. The first limitation is the small sample size and that only images from healthy volunteers were acquired, so it remains to be seen how these findings translate into the clinical evaluation of patients. However, vessel wall lesions generally thicken the wall, so it is believed that the IV-SPACE sequence will have a clearer depiction of the LSA wall. Another limitation is that collecting the field map and designing RF pulse for each subject is time-consuming, so some general pulse design methods should be considered for further study.

## Conclusion

The inner-volume 3D TSE sequence was developed to achieve isotropic 0.30 mm within ten minus black-blood images within a clinically acceptable time. A higher resolution produces sharper delineation of the vessel wall and lumen of intracranial perforating arteries. The technique is promising for the evaluation of microvasculopathies of cerebral vascular diseases.

## Data Availability Statement

The raw data supporting the conclusions of this article will be made available by the authors, without undue reservation.

## Ethics Statement

The studies involving human participants were reviewed and approved by the Institutional Review Board of Beijing MRI Center for Brain Research. The patients/participants provided their written informed consent to participate in this study.

## Author Contributions

ZZ and QK: study concepts and design. QK, DW, and ZZ: sequence programming. QK: sequence test. QK and YW: data acquisition. QK, YW, and ZZ: data analysis and interpretation. QK: manuscript preparation. ZZ, YZ, and JA: manuscript editing. YZ and ZZ: manuscript review. All authors contributed to the article and approved the submitted version.

## Conflict of Interest

QK and JA are employees of Siemens Healthcare, China. DW is an employee of Siemens Shenzhen Magnetic Resonance Ltd. The remaining authors declare that the research was conducted in the absence of any commercial or financial relationships that could be construed as a potential conflict of interest.

## Supplementary Material

Supplementary Figure 1 The line profile through the center of ROI before the normalization. The solid line is for the optimized pulse and the dashed line is the traditional pulse.
