# Supplementary figures and images for: Optimized Inner-Volume 3D TSE for High-Resolution Vessel Wall Imaging of Intracranial Perforating Arteries at 7T
Source: Front Neurosci. 2021 Feb 25;15:620172. doi: 10.3389/fnins.2021.620172 (PMC7947629; doi:10.3389/fnins.2021.620172)

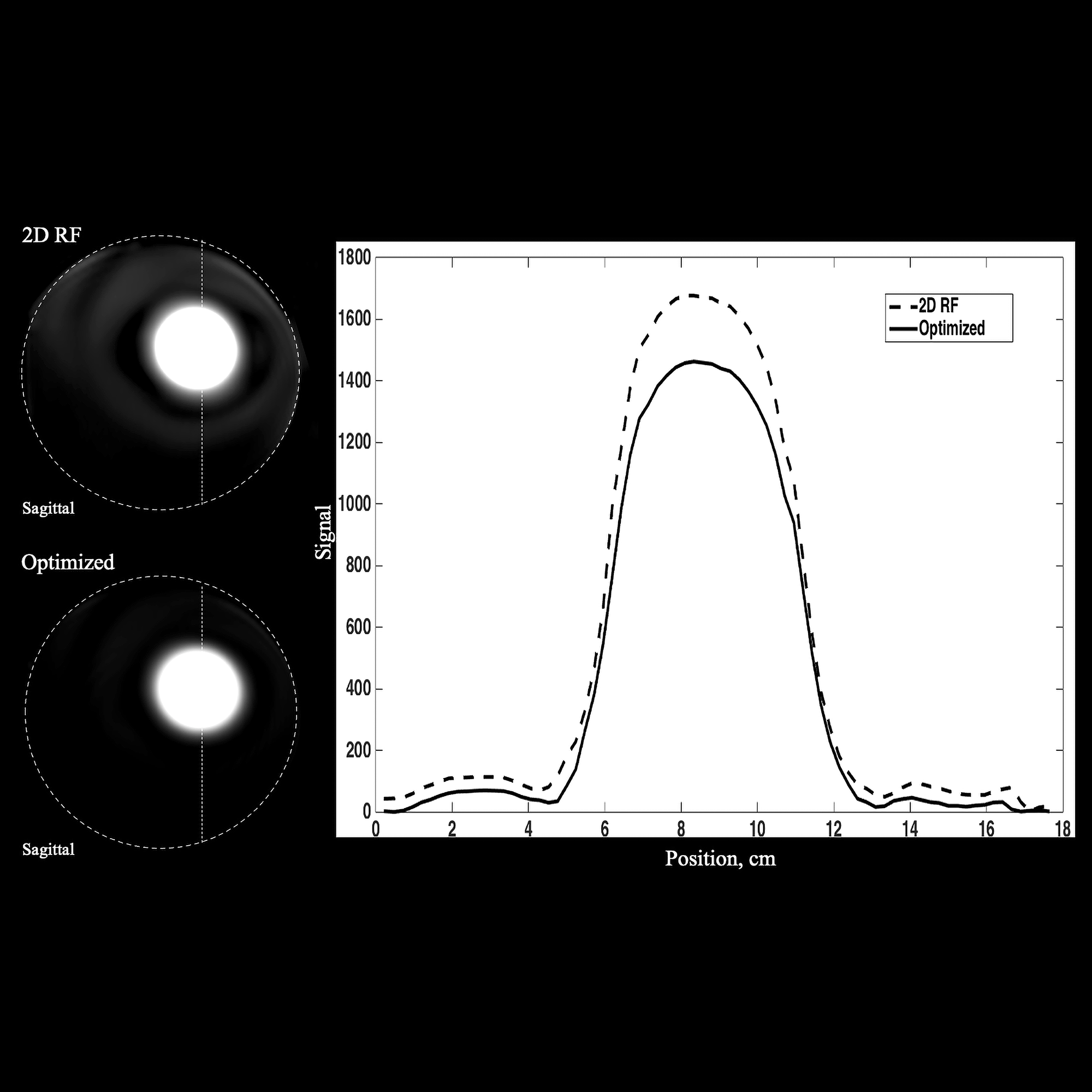

Supplement: Supplementary Figure 1 — The line profile through the center of ROI before the normalization. The solid line is for the optimized pulse and the dashed line is the traditional pulse. [file Image_1.TIF]
